# The Leaf Essential Oil of *Gynoxys buxifolia* (Kunth) Cass. (Asteraceae): A Good Source of Furanoeremophilane and Bakkenolide A

**DOI:** 10.3390/plants12061323

**Published:** 2023-03-15

**Authors:** Carolina Cumbicus, Omar Malagón, Nixon Cumbicus, Gianluca Gilardoni

**Affiliations:** 1 Departamento de Química, Universidad Técnica Particular de Loja (UTPL), Calle Marcelino Champagnat s/n, Loja 110107, Ecuador; mccumbicus2@utpl.edu.ec (C.C.); omalagon@utpl.edu.ec (O.M.); 2 Departamento de Ciencias Biológicas y Agropecuarias, Universidad Técnica Particular de Loja (UTPL), Calle Marcelino Champagnat s/n, Loja 110107, Ecuador; nlcumbicus@utpl.edu.ec

**Keywords:** *Gynoxys buxifolia*, essential oil, GC-MS, enantioselective analysis, furanoeremophilane, bakkenolide A

## Abstract

The present study describes the chemical and enantiomeric composition of a new essential oil, distilled from the dry leaves of *Gynoxys buxifolia* (Kunth) Cass. The chemical analysis was conducted by GC-MS and GC-FID, on two orthogonal capillary columns. A total of 72 compounds were detected and quantified with at least one column, corresponding to about 85% by weight of the whole oil mass. Of the 72 components, 70 were identified by comparing the respective linear retention indices and mass spectra with data from the literature, whereas the two main constituents were identified by preparative purification and NMR experiments. The quantitative analysis was carried out calculating the relative response factor of each compound according to their combustion enthalpy. The major constituents of the EO (≥3%) were: furanoeremophilane (31.3–28.3%), bakkenolide A (17.6–16.3%), caryophyllene oxide (6.0–5.8%), and (*E*)-β-caryophyllene (4.4%). Additionally, the hydrolate was also analyzed with respect to the dissolved organic phase. About 40.7–43.4 mg/100 mL of organic compounds was detected in solution, of which *p*-vinylguaiacol was the main component (25.4–29.9 mg/100 mL). Finally, the enantioselective analysis of some chiral terpenes was carried out, with a capillary column based on β-cyclodextrin chiral stationary phase. In this analysis, (1*S*,5*S*)-(−)-α-pinene, (1*S*,5*S*)-(−)-β-pinene, (*S*)-(+)-α-phellandrene, (*S*)-(+)-β-phellandrene, and (*S*)-(−)-terpinen-4-ol were detected as enantiomerically pure, whereas (*S*)-(−)-sabinene showed an enantiomeric excess of 69.2%. The essential oil described in the present study is a good source of two uncommon volatile compounds: furanoeremophilane and bakkenolide A. The former lacks bioactivity information and deserves further investigation, whereas the latter is a promising selective anticancer product.

## 1. Introduction

Essential oils (EOs) are defined by the European Pharmacopoeia as “odorous products, usually of complex composition, obtained from a botanically defined plant raw material by steam-distillation, dry distillation, or a suitable mechanical process without heating” [1]. Since these products present a wide range of applications and biological activities, together with a great commercial interest, our group is currently engaged in the study of volatile fractions from plants [2,3,4,5,6,7,8]. In particular, we are interested in EOs characterized by the presence of abundant sesquiterpene fractions, possibly dominated by new or rare sesquiterpenes. For ecological reasons, such a research line, as well phytochemistry in general, is quite promising in Ecuador. In fact, Ecuador is mentioned among the so-called “megadiverse” countries, a group of 17 countries hosting three-fourths of all higher plant species of the world, most of which remain unstudied [9,10,11]. According to some preliminary unpublished analyses, the genus *Gynoxys* (Asteraceae) is an excellent candidate for a systematic investigation in this sense. Though the distillation yield is usually quite low, these EOs are rich in metabolites, characterized by sesquiterpenes and, as the present study demonstrates, sometimes dominated by few uncommon major compounds. Therefore, this work is part of an unfunded project, whose aim is the description of the EOs, obtained from the species of the genus *Gynoxys* growing in the province of Loja (Ecuador). This systematic research began with the recent publications about the volatile fractions in the leaves of *G. miniphylla* and *G. rugulosa* and continues with the description of EOs from other species currently under investigation [12,13].

For what concerns the species *G. buxifolia*, only two studies have been published so far: one focusing on ecology, and the other covering the phytochemistry of the non-volatile fraction [14,15]. To the best of the authors’ knowledge, no information has been reported about the EO. From the botanical point of view, *Gynoxys buxifolia* (Kunth) Cass. is a shrub or treelet, native to the Andean region, and growing between 3000–4000 m above sea level [16]. Furthermore, this species is known by three synonyms: *Eupatorium bicolor* Lam. ex DC., *Gynoxys buxifolia* var. *brevifolia* Hieron., and *Senecio buxifolius* Kunth [17]. With respect to the geographical distribution, *G. buxifolia* is almost exclusive to Ecuador, with a few exceptions in Peru and Colombia [16]. This plant, whose traditional name is *Tucshi*, does not possess medicinal applications; however, the leaves are used as forage for sheep and guineapigs [18].

## 2. Results

### 2.1. Chemical Composition of the EO and Hydrolate

In the chemical analysis of the EO and hydrolate of *G. buxifolia*, a total of 72 compounds were detected and identified with at least one gas chromatographic column.

Regarding the EO, it is dominated by the sesquiterpene fraction, corresponding to 79.4–76.2% of the whole EO mass, with a non-polar and polar column. Furthermore, the monoterpene fraction accounted for 5.9–5.7% of the entire oil. Altogether, the quantified constituents corresponded to 87.9–84.3% of the whole EO. The main components in the volatile fraction of *G. buxifolia* (≥3.0%) were furanoeremophilane (**67**, 31.3–28.3%), bakkenolide A (**72**, 17.6–16.3%), caryophyllene oxide (**61**, 6.0–5.8%), and (E)-β-caryophyllene (**39**, 4.4%). Almost all the components of the EO were identified by mass spectrum and linear retention index (LRI), except for the rare sesquiterpenes furanoeremophilane (**67**) and bakkenolide A (**72**), whose identification was carried out through nuclear magnetic resonance (NMR) spectroscopy, with ^1^H and ^13^C NMR experiments. After comparing the MS and NMR spectral data with those from literature, the main constituent of the EO appeared to be identical to furanoeremophilane (**67**, 114.3 mg), whereas the second main constituent was identical to bakkenolide A (**72**, 6.6 mg) [19,20,21,22,23,24]. The original NMR and MS spectra are reported in the Supplementary Materials.

**Figure 1:**
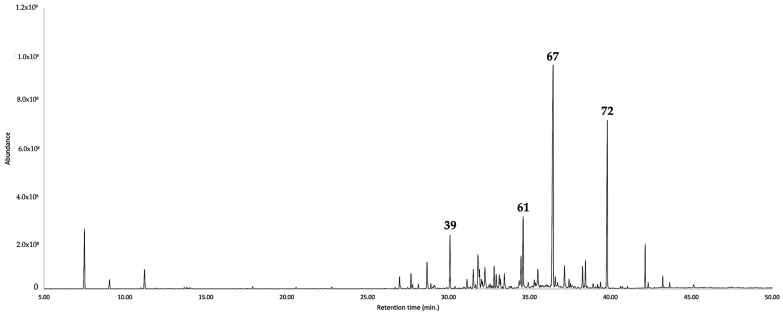
GC-MS chromatogram of the leaf EO of *G. buxifolia* in a 5%-phenyl-methylpolysiloxane-based column. The main components are represented with the respective peak number, according to Table 1.

**Figure 2:**
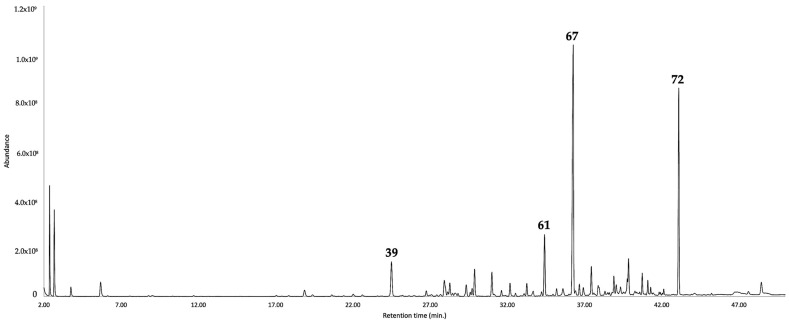
GC-MS chromatogram of the leaf EO of *G. buxifolia* in a polyethylen glicol-based column. The main components are represented with the respective peak number, according to Table 1.

**Figure 3:**
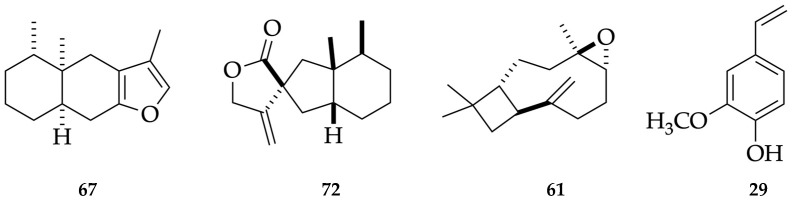
Main compounds identified in the leaf EO and hydrolate of *G. buxifolia*: furanoeremophilane (**67**), bakkenolide A (**72**), caryophyllene oxide (**61**), and *p*-vinylguaiacol (**29**).

For what concerns the hydrolate, the chemical analysis was expressed as milligrams of organic compounds per 100 mL of water. The total amount of organic substances corresponded to 40.7–43.4 mg/100 mL, entirely constituted of oxygenated molecules. Though, in the EO, the non-terpene components accounted for only 2.6–2.4%, in the hydrolate they corresponded to about 75% of the organic fraction. The main constituent in the water phase was *p*-vinylguaiacol (**29**), corresponding to more than 50% of the organic fraction in solution. Of all the metabolites, (2*E*,4*E*)-heptadienal, ο-tolualdehyde, linalool oxide (furanoid), *p*-mentha-1,5-dien-8-ol, 2-allylphenol, *p*-vinylguaiacol, and eremophilone were only present in the aqueous phase, whereas 1,8-cineole, linalool, terpinen-4-ol, γ-terpineol, caryophyllene oxide, cyclocolorenone, and bakkenolide A were detected in both phases. The results of the chemical analyses are detailed in Table 1 as well as Figure 1 and Figure 2, whereas the major components are represented in Figure 3.

### 2.2. Enantioselective Analysis of the EO

The EO was submitted to enantioselective analysis, through a 2,3-diacetyl-6-tert-butyldimethylsilyl-β-cyclodextrin-based column. Seven quiral terpenes were detected, and their enantiomeric distribution analyzed by comparing the respective linear retention indices with those calculated for enantiomerically pure standards. Only sabinene appeared as an enantiomeric pair, with the levorotatory isomer showing an enantiomeric excess (*e.e.*) of 69%. All the other chiral metabolites were enantiomerically pure, whereas the optical isomers of limonene are inseparable with this chiral selector. The detailed results of the enantioselective analysis are reported in Table 2.

## 3. Discussion

The chemical analyses of the EO and hydrolate were carried out through two ortogonal columns. The results are qualitatively and quantitatively comparable, with the polar column confirming most of the analytes, detected and quantified with the non-polar stationary phase. Practically, all the constituents detected as traces in the EO with the polar column were compounds close to the detection threshold on the non-polar phase. This fact resulted in a little lower total amount with the polyethylen glycol column with respect to the polysiloxane stationary phase, being 84.3% vs. 87.9%, respectively. This difference (3.6%) is spread over 72 analytes, resulting in a mean value of 0.05% for each compound. Similar considerations can be made for the hydrolate. Therefore, despite the small differences, the qualitative and quantitative analyses of both columns can be considered reciprocally consistent.

The main goal of the present study, apart from the description of an unprecedented EO and its hydrolate, is the identification of *G. buxifolia* volatile fraction as a good source of furanoeremophilane (**67**) and bakkenolide A (**72**). These compounds are well known, but the experience demonstrates that they are quite uncommon in the EOs, and even less so as the two main constituents (about 30% and 17% by weight, respectively, in the present EO). Furanoeremophilane (**67**) was discovered in 1961 by Novotny et al. in the rhizomes of *Petasites officinalis* and *P. albus*, together with some derivatives and other related sesquiterpenes [68,69,70]. Subsequently, its structure was spectroscopically confirmed through NMR experiments by other authors [19,20,21,22,23]. More recently, compound **67** and other furanoeremophilanes have been described in different botanical species, such as *Lopholaena dregeana*, *L. platyphylla*, *Bedfordia salicina*, *Senecio inornatus*, *S. halimifolius*, *S. medley-woodii*, *S. inaequidens*, and *P. hybridus,* among others [71,72,73,74]. Sometimes, non-volatile furanoeremophilanes have been found in fixed fractions through solvent extraction. This is the case of the only existing phytochemical study about *G. buxifolia*, where 15 non-volatile furanoeremophilanes were identified in *G. acostae*, *G. buxifolia*, and *G. nitida* [15].

**Figure 4:**
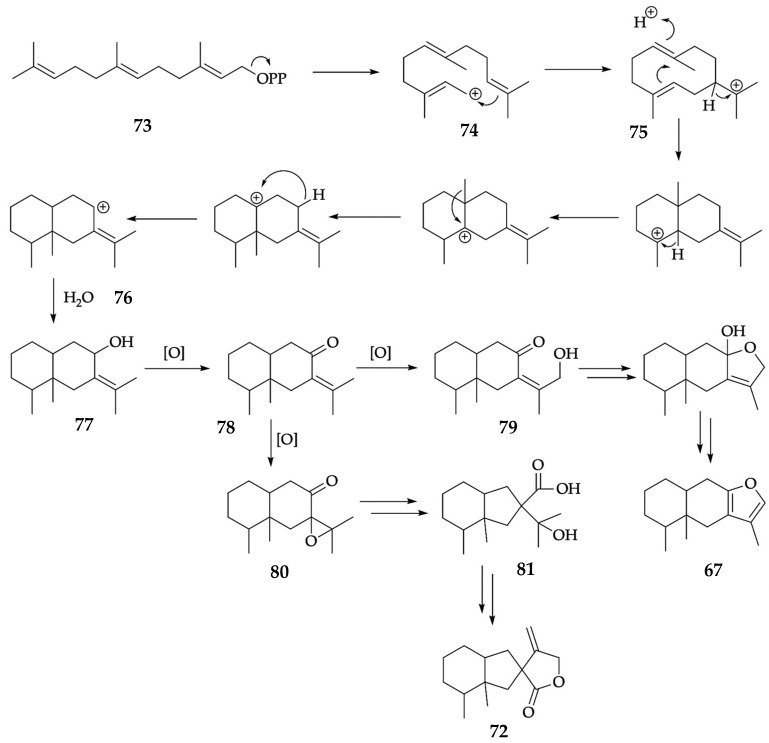
Hypothetical biosynthesis proposed for furanoeremophilane (**67**) and bakkenolide A (**72**).

Bakkenolide A (**72**) was discovered in 1968 in *P. japonicus*, a plant from the same genus in which furanoeremophilane (**67**) had been discovered a few years before [22,23,75]. Compound **72** was then identified as a typical metabolite of *P. albus* itself and, more recently, the same compound and 51 structurally related derivatives were discovered in other species of the same genus [76,77,78]. Bakkenolide A (**72**) has been found in some species from genera *Ligularia*, *Homogyne*, *Cacalia*, *Cetraria*, and *Hertia* [79,80,81,82]. It is interesting to observe that, according to the literature, furanoeremophilane (**67**) and bakkenolide A (**72**) were often found in the same plants, suggesting a common biosynthesis for these oxygenated sesquiterpenes. Though the literature usually presents only partial biosynthetic pathways for compounds **67** and **72**, a quite complete scheme can be suggested, as in Figure 4.

As usual, the allylic farnesyl cation (**74**) proceeds from farnesyl pyrophosphate (**73**), the common precursor of all sesquiterpenes. After that, the intramolecular nucleophilic addition of the terminal double bond to the allyl carbocation affords the germacryl cation (**75**). At this point, three subsequent carbocation transpositions produce the intermediate **76**, which is converted to hydroxy eremophilane (**77**) by the addition of a molecule of water. Compound **77** undergoes oxidation to fukinone (**78**) which, through further oxidation, can be converted to the hydroxy fukinone **79** or to the fukinone epoxide **80**. The intermediate **79**, in a few steps, affords furanoeremophilane (**67**), whereas **80**, after a Favorskii-like rearrangement, produces the hydroxy acid **81**. Finally, compound **81**, after dehydration and few more steps, produces bakkenolide A (**72**) [83,84,85].

For what concerns the biological activities of these major compounds, bakkenolide A (**72**) is clearly the most interesting. In fact, though many furanoeremophilanes have been described for possessing important biological properties, no relevant studies have been found about compound **67**. On the other hand, the literature is rich in publications about bakkenolide A (**72**) and its biological capacities. There are two main properties of this metabolite: anticancer and antifeedant. The anticancer property was first described in 1976, when compound **72** was discovered to be cytotoxic against human and rodent cells [77]. These results were extremely promising, since bakkenolide A (**72**) appeared to be more toxic against human than rodent cells. Furthermore, the activity against cancerous cells was five times higher than the one against normal cells. This evidence suggested a selective cytotoxicity against human cancer, which is the main feature of an ideal anticancer drug. More recently, bakkenolide A (**72**) was discovered to be active against leukemia, by inhibiting the synthesis of histone deacetylase (HDAC3) [86]. This enzyme is responsible for the acetylation of proteins, and its activity is known to work improperly in human cancer. Furthermore, in leukemia cells, HDAC3 is overexpressed. For these reasons, the inhibitors of HDAC3 are considered to be potential anticancer drugs. In the cited study, bakkenolide A (**72**) is not a direct HDAC3 inhibitor, but it was shown to reduce the enzyme expression. The underexpression of HDAC3 passes through the inhibition of IκBα, producing the deactivation of NF-κB and, therefore, the suppression of inflammation. As a result, apoptosis is enhanced, cancer diffusion is reduced, and an indirect cytotoxic effect against healthy cells is observed [86].

The second important biological activity of compound **72** was demonstrated in many studies. In fact, bakkenolide A (**72**) is a powerful antifeedant compound against *Sitophilus granarius*, *Tribolium confusum*, *Trogoderma granarium*, and *Peridroma saucia* [87,88].

Since the EO of *G. buxifolia* spontaneously separated from the aqueous phase, the hydrolate constitutes an interesting byproduct, suitable for investigation. The leaf hydrolate was dominated by the presence of *p*-vinylguaiacol (**29**), corresponding to about 67% by weight of the dissolved organic fraction and providing water a pleasant aroma. In 2014, *p*-vinylguaiacol (**29**) was investigated as the main degradation product of curcumin while cooking. As a result, compound **29** showed no cytotoxic properties, but it was effective as an activator of the transcription factors Nrf2 and PON1 in a dose-dependent manner. Furthermore, it produced the downregulation of interleukin-6 mRNA levels in a stain of murine macrophages. Therefore, *p*-vinylguaiacol (**29**) can be considered a non-cytotoxic, antioxidant and anti-inflammatory product [89]. On this basis, the leaf hydrolate of *G. buxifolia* could be potentially used as an aqueous phase in the formulation of nutraceuticals and cosmeceuticals.

Finally, the chemical description of this EO could not be considered exhaustive without the enantioselective analysis of at least some chiral components. To the best of the authors’ knowledge, there are only two EOs from the genus *Gynoxys* whose enantioselective analyses are currently available in literature: *G. miniphylla* and the volatile fraction from *G. rugulosa* [12,13]. From the comparison between the three volatile fractions, a great difference in the enantiomeric composition emerged. In particular, we can observe that in *G. miniphylla* and *G. rugulosa*, most of the investigated chiral compounds were present as scalemic mixtures, with enantiomeric excesses that differ in between the two species. On the other hand, *G. buxifolia* EO was dominated by enantiomerically pure constituents. Another point was the opposite absolute configuration of the dominant enantiomers. For example, α-phellandrene and β-phellandrene were 100% levorotatory in *G. miniphylla* and 100% dextrorotatory in *G. buxifolia*. All these differences could be the result of climatic or ecological factors; however, they demonstrate the existence of different biosynthetic pathways, devoted to the biosynthesis of different enantiomers, produced by the plant for different functions [85]. In fact, despite being two enantiomers characterized by the same chemical-physical properties, they differ in a biological medium for their physiological and pharmacological activities. In the case of the EOs, whose main property is the aroma, two optical isomers of the same chiral metabolite can present different olfactory properties [90]. For this reason, enantioselective analysis is nowadays a fundamental aspect of an EO description, since different volatile fractions, characterized by similar conventional chromatographic profiles, can present a very different aroma due to their various enantiomeric compositions.

## 4. Materials and Methods

### 4.1. Chemicals, Materials, and Equipment

All GC analyses were carried out with a Trace 1310 gas chromatograph (Thermo Fisher Scientific, Walthan, MA, USA). For the qualitative and enantioselective analyses, the instrument was coupled to a simple quadrupole mass spectrometry detector, model ISQ 7000, also from Thermo Fisher Scientific, whereas for the quantitative analyses, a flame ionization detector (FID) was applied. The mass spectrometer was operated in SCAN mode (scan range 40–400 *m*/*z*), with the electron ionization (EI) source set at 70 eV. The ion source and transfer line were programmed at 230 °C. Two orthogonal capillary columns were used for both the qualitative and quantitative analyses: a non-polar column, based on 5%-phenyl-methylpolysiloxane (DB-5ms), and a polar one (HP-INNOWax), based on polyethylene glycol. Both columns were 30 m long, with 0.25 mm internal diameter and 0.25 μm film thickness (Agilent Technology, Santa Clara, CA, USA). The enantioselective analysis was carried out through a 2,3-diacetyl-6-*tert*-butyldimethylsilyl-β-cyclodextrin-based capillary column (25 m × 250 μm internal diameter × 0.25 μm phase thickness), purchased from Mega, MI, Italy. The carrier gas was GC purity grade helium, purchased from Indura, Guayaquil, Ecuador. All NMR experiments were carried out with a 500 MHz Bruker spectrometer (Bruker, Billerica, MA, USA), whereas deuterated solvents were purchased from Sigma-Aldrich (St. Louis, MO, USA). The preparative chromatographic separations on column were carried out with a Reveleris^®®^ PREP Purification System, equipped with commercial normal phase (silica gel 60) packed columns and with both a UV-vis and a light scattering detector. The chromatograph and columns were purchased from Büchi (Büchi Labortechnik, Flawil, Switzerland). The thin-layer chromatography separations (TLC), both analytical and preparative, were conducted over silica gel 60 (0.25 mm; GF_254_, Merck) plates (from Sigma-Aldrich). After elution, the TLC plates were initially visualized under UV light (254 and 366 nm), then sprayed with a 0.5% solution of vanillin in H_2_SO_4_/ethanol 4:1, and finally heated at 200 °C. For all the GC analyses, analytical purity grade solvents were used, whereas for analytical TLC and other preparative applications, reagent grade solvents were applied. For the solid phase extraction (SPE), the cartridges were standard products, packed with 1 g of C18 reversed phase and purchased from Sigma-Aldrich. All the solvents, *n*-alkanes (C_9_–C_22_) for retention indices, and internal standard (*n*-nonane) were purchased from Sigma-Aldrich. The calibration standard for GC-FID analyses was isopropyl caproate, obtained by synthesis in the authors’ laboratory and purified to 98.8% (GC-FID).

### 4.2. Plant Material

The leaves of *G. buxifolia* were collected on 22 September 2021 from shrubs located in a range of 200 m around a central point, of coordinates 04°22′58″ S and 79°09′01″ W. The collection area is situated in the parish of Yangana, province of Loja, at the altitude of 2800 m above sea level. The botanical identification was carried out by one of the authors (N.C.), by means of the herbarium specimens with US Catalog No.: 3539375 Barcode: 01826039, conserved at the National Museum of Natural History, Smithsonian Institution. A botanical sample of the collected plant was also deposited at the herbarium of the UTPL, with voucher code 14669. After collection, the plant was dried at 35 °C for 48 h, affording 2.9 kg of dry leaves as a mean sample, obtained from many different shrubs. This investigation was carried out under permission of the Ministry of Environment, Water and Ecological Transition of Ecuador, with MAATE registry number MAE-DNB-CM-2016-0048.

### 4.3. Obtention of the EO and Hydrolate

The entire amount of dry plant (2.9 kg) was submitted to steam-distillation for 4 h, in a stainless-steel Clevenger-type apparatus, producing a dark aromatic EO that spontaneously separated from water. The yield of EO was calculated as 0.1% by weight with respect to the dry plant material. Additionally, 50 mL of hydrolate was collected for the analysis of the dissolved organic fraction. Both EO and water phase were stored at −15 °C until use.

### 4.4. GC Sample Preparation

Four samples were prepared by exactly weighing about 10 mg of EO and diluting them with exactly 1 mL of cyclohexane, spiked with *n*-nonane as an internal standard. The concentration of *n*-nonane was 0.7 mg/mL. For the hydrolate, four samples were prepared by passing through the reversed phase SPE cartridges, previously washed with methanol and re-conditioned with water and an exact volume of 10 mL of hydrolate. After complete removal of the water phase, the cartridge was eluted with 2 mL of acetone spiked with *n*-nonane as internal standard. The concentration of *n*-nonane in acetone was the same as in cyclohexane. Both the EO and hydrolate samples were then directly injected in GC.

### 4.5. Qualitative GC-MS Analyses

The qualitative analyses were conducted in GC-MS by injecting 1 μL of sample with both polar and non-polar columns (spilt ratio 40:1). In all cases, the carrier gas (He) was set at the constant flow of 1 mL/min, whereas the thermal program was as follows: 50 °C for 5 min, followed by a thermal gradient of 3 °C/min until 100 °C, then a second gradient of 5 °C/min until 180 °C, and a final ramp of 10 °C/min until 230 °C. The final temperature was maintained for 5 min. A mixture of *n*-alkanes, from C_9_ to C_22_, was also injected under the same conditions, in order to calculate the linear retention indices (LRIs) of the sample constituents, according to van den Dool and Kratz [91]. The qualitative composition of the EO and hydrolate was determined by comparing the mass spectra and LRIs with data from literature (see Table 1).

### 4.6. Quantitative GC-FID Analyses

The quantitative analyses were conducted in GC-FID, with the same method, columns, and instrument configuration as GC-MS. All the analytes were quantified according to the principle that, in GC-FID, the relative response factors (RRFs) only depend on the combustion enthalpy of each compound and can be mathematically calculated [92,93]. After applying the RRFs to all the integration areas, a calibration curve was traced for each column, using isopropyl caproate as a calibration standard. The dilutions for the construction of the calibration curves were prepared as previously described in the literature, obtaining a correlation coefficient of 0.9995 [7].

### 4.7. Enantioselective Analysis of the EO

The enantioselective analysis was carried out in GC-MS, with the chiral-phase column described in Section 4.1 and the same instrument configuration as the qualitative analyses. The thermal program was as follows: 50 °C for 5 min, followed by a thermal gradient of 2 °C/min until 220 °C, which was maintained for 5 min. The same mixture of *n*-alkanes used for the qualitative analyses was also injected, and the LRIs calculated. The enantiomers of the chiral compounds, separable with this chiral selector, were identified for possessing the same mass spectrum and by comparing their LRIs with those of enantiomerically pure standards.

### 4.8. Purification and Identification of Furanoeremophilane and Bakkenolide A

To purify furanoeremophilane (**67**) and bakkenolide A (**72**), 1 g of pure EO was loaded onto a 220 g silica gel column and fractionated by means of the automatic chromatograph described in Section 4.1. The elution was carried out with a mixture of petroleum ether/diethyl ether, according to an increasing gradient of polarity and with a constant flow of 120 mL/min. Starting from pure petroleum ether, which was maintained for 2.5 min, the percentage of diethyl ether was increased by 2% each minute until 80% petroleum ether, which was maintained for 2 min. The diethyl ether was then raised to 25%, maintained for 5 min, followed by a new polarity gradient where the diethyl ether increased by 10% every 2 min. The gradient ended when the composition of the mobile phase reached 80% diethyl ether. This process produced 130 fractions of different volumes, which were submitted to TLC analysis (petroleum ether/diethyl ether 80:20). The tubes of similar composition were reunited, and the solvent evaporated at reduced pressure, finally obtaining 12 fractions (A1–A12 in order of increasing polarity). To identify the fractions containing the two major components, the 12 samples were injected in GC-MS with the non-polar column, under the same conditions as the qualitative analysis. On the one hand, the fraction A1 contained the first major compound, corresponding to peak **67**, in an almost pure form, and it was directly submitted to ^1^H and ^13^C NMR. On the other hand, the second major component, corresponding to peak **72**, was detected in fractions A8–A10, as a mixture with other compounds. Therefore, the three fractions were reunited, and 20 mg was fractionated on normal phase preparative TLC, eluting with a mixture of petroleum ether/dichloromethane/methanol in the ratio 50:45.5:0.5. The purified metabolite was submitted to ^1^H and ^13^C NMR spectroscopy.

## 5. Conclusions

The leaves of *Gynoxys buxifolia* (Kunth) Cass. produce an EO and an hydrolate, which are described in this study for the first time. Two biosynthetically related uncommon oxygenated sesquiterpenes are the main constituents of the EO: furanoeremophilane and bakkenolide A. Thanks to the good yield, the high amount of the two components, and the easiness of their purification, this EO is a good source of both metabolites for further bioactivity research. Furthermore, the enantioselective analysis supported the existence of different biosynthetic pathways, to produce different enantiomers in this and other EOs. Finally, the presence of *p*-vinylguaiacol as the major organic component of the hydrolate has been described, together with a possible application.

## Figures and Tables

**Table 1:** Qualitative and quantitative analyses of *G. buxifolia* EO and hydrolate.

N.	Compound	5%-Phenyl-Methylpolysiloxane							Polyethylene Glycol						
		LRI ^1^	LRI ^2^	Reference	Essential Oil		Hydrolate		LRI ^1^	LRI ^2^	Reference	Essential Oil		Hydrolate	
					%	σ	mg/100 mL	σ				%	σ	mg/100 mL	σ
**1**	α-pinene	933	932	[25]	1.1	0.01	-	-	1015	1022	[26]	1.3	0.10	-	-
**2**	sabinene	974	969	[25]	0.1	0.01	-	-	1115	1120	[27]	trace	-	-	-
**3**	β-pinene	979	974	[25]	2.7	0.05	-	-	1102	1105	[28]	2.6	0.17	-	-
**4**	myrcene	992	988	[25]	trace	-	-	-	1161	1161	[29]	trace	-	-	-
**5**	*n*-decane	1000	1000	[25]	0.1	0.01	-	-	1000	-	*	trace	-	-	-
**6**	α-phellandrene	1009	1002	[25]	trace	-	-	-	1156	1164	[30]	trace	-	-	-
**7**	(2*E*,4*E*)-heptadienal	1024	1017	[25]	-	-	0.4	0.03	1484	1492	[31]	-	-	0.5	0.06
**8**	*ο*-cymene	1029	1022	[25]	0.1	0.01	-	-	1262	1266	[32]	trace	-	-	-
**9**	limonene	1032	1024	[25]	0.1	0.01	-	-	1190	1197	[30]	trace	-	-	-
**10**	β-phellandrene	1034	1025	[25]	0.1	0.01	-	-	1197	1195	[33]	trace	-	-	-
**11**	1,8-cineole	1036	1026	[25]	0.1	0.01	0.6	0.13	1195	1190	[34]	trace	0.01	1.1	0.15
**12**	*ο*-tolualdehyde	1058	1068	[35]	-	-	0.6	0.06	1638	1636	[36]	-	-	0.2	0.23
**13**	γ-terpinene	1061	1054	[25]	trace	-	-	-	1244	1244	[37]	trace	-	-	-
**14**	linalool oxide (furanoid)	1075	1067	[25]	-	-	1.9	0.56	1436	1439	[38]	-	-	0.7	0.32
**15**	*p*-mentha-2,4(8)-diene	1088	1085	[25]	trace	-	-	-	1287	1286	[39]	trace	-	-	-
**16**	linalool	1106	1095	[25]	0.1	0.01	1.0	0.08	1553	1554	[40]	trace	-	1.0	0.29
**17**	*n*-nonanal	1112	1100	[25]	0.4	0.01	-	-	1388	1387	[28]	trace	-	-	-
**18**	*trans*-*p*-menth-2-en-1-ol	1150	1136	[25]	0.1	0.05	-	-	1604	1609	[41]	0.1	0.05	-	-
**19**	ethyl benzoate	1167	1169	[25]	0.2	0.01	-	-	-	-	-	-	-	-	-
**20**	*p*-mentha-1,5-dien-8-ol	1184	1185	[42]	-	-	0.8	0.10	1724	1725	[43]	-	-	0.7	0.06
**21**	terpinen-4-ol	1188	1174	[25]	0.1	0.01	1.3	0.09	1594	1595	[44]	trace	-	1.6	0.52
**22**	*n*-dodecane	1200	1200	[25]	0.1	0.05	-	-	-	-	-	-	-	-	-
**23**	2-allylphenol	1199	1189	[25]	-	-	0.8	0.08	-	-	-	-	-	-	-
**24**	γ-terpineol	1205	1199	[25]	trace	-	1.4	0.06	1692	1696	[45]	trace	-	0.9	0.17
**25**	*n*-decanal	1215	1201	[25]	0.3	0.01	-	-	1493	1502	[46]	trace	-	-	-
**26**	geraniol	1262	1249	[25]	trace	-	-	-	1850	1851	[47]	0.4	0.03	-	-
**27**	carvacrol	1312	1298	[25]	0.9	0.05	-	-	2196	2189	[48]	1.3	0.10	-	-
**28**	silphiperfol-5-ene	1325	1326	[25]	trace	0.01	-	-	1407	1407	[33]	-	-	-	-
**29**	*p*-vinylguaiacol	1326	1309	[49]	-	-	25.4	1.33	2196	2196	[50]	-	-	29.9	1.15
**30**	presilphiperfol-7-ene	1336	1334	[25]	0.2	0.01	-	-	-	-	-	-	-	-	-
**31**	undetermined (mw = 204)	1347	-	-	0.9	0.05	-	-	1432	-	-	1.3	0.10	-	-
**32**	7-*epi*-silphiperfol-5-ene	1350	1345	[25]	0.1	0.05	-	-	1444	1454	[51]	trace	-	-	-
**33**	silphiperfol-5,7(14)-diene	1361	1358	[25]	0.3	0.05	-	-	1509	1523	[51]	trace	-	-	-
**34**	α-copaene	1378	1374	[25]	trace	-	-	-	1525	1525	[52]	trace	-	-	-
**35**	geranyl acetate	1385	1379	[25]	0.7	0.01	-	-	1756	1752	[51]	1.2	0.26	-	-
**36**	β-cubebene	1391	1387	[25]	0.2	0.01	-	-	1526	1522	[53]	trace	-	-	-
**37**	β-elemene	1393	1389	[25]	0.4	0.01	-	-	1561	1563	[33]	0.1	0.15	-	-
**38**	*n*-tetradecane	1400	1400	[25]	0.1	0.01	-	-	1400	-	*	trace	-	-	-
**39**	(*E*)-β-caryophyllene	1425	1417	[25]	4.4	0.06	-	-	1578	1572	[54]	4.4	0.40	-	-
**40**	γ-elemene	1432	1434	[25]	0.1	0.01	-	-	1645	1644	[55]	trace	-	-	-
**41**	α-funebrene	1436	1438	[56]	0.1	0.05	-	-	1529	-	*	trace	-	-	-
**42**	*cis*-cadina-1(6),4-diene	1456	1461	[25]	0.1	0.01	-	-	1558	-	*	0.1	0.15	-	-
**43**	α-humulene	1463	1452	[25]	0.8	0.06	-	-	1651	1650	[57]	1.2	0.19	-	-
**44**	*cis*-muurola-4(14),5-diene	1471	1465	[25]	0.1	0.01	-	-	1657	-	*	0.1	0.15	-	-
**45**	α-neocallitropsene	1482	1474	[25]	0.2	0.10	-	-	-	-	*	trace	-	-	-
**46**	ar-curcumene	1487	1479	[25]	1.5	0.05	-	-	1766	1763	[51]	1.4	0.22	-	-
**47**	*trans*-muurola-4(14),5-diene	1489	1493	[25]	2.8	0.08	-	-	1691	-	*	2.5	0.24	-	-
**48**	*cis*-β-guaiene	1490	1492	[25]	2.1	0.10	-	-	1704	1702	[58]	2.4	0.38	-	-
**49**	valencene	1495	1496	[25]			-	-	1691	1689	[54]	2.5	0.24	-	-
**50**	β-selinene	1497	1489	[25]			-	-	1698	1698	[59]	0.1	0.25	-	-
**51**	β-himachalene	1513	1510	[60]	0.4	0.01	-	-	1622	1632	[51]	0.1	0.10	-	-
**52**	(*Z*)-γ-bisabolene	1516	1514	[25]	0.3	0.06	-	-	1878	-	*	trace	-	-	-
**53**	*n*-tridecanal	1519	1509	[25]	0.3	0.05	-	-	-	-	-	-	-	-	-
**54**	γ-cadinene	1521	1513	[25]	0.3	0.01	-	-	1716	1716	[61]	0.1	0.15	-	-
**55**	δ-cadinene	1525	1522	[25]	0.3	0.01	-	-	1745	1745	[62]	0.1	0.25	-	-
**56**	zonarene	1531	1528	[25]	1.3	0.01	-	-	1760	-	*	1.2	0.22	-	-
**57**	undetermined (mw = 202)	1541	-	-	0.7	0.01	-	-	1863		-	0.4	0.03	-	-
**58**	italicene ether	1546	1536	[25]	0.3	0.01	-	-	1845	1830	[63]	0.4	0.03	-	-
**59**	β-vetivenene	1551	1554	[25]	0.2	0.01	-	-	2074	-	*	0.1	0.15	-	-
**60**	spathulenol	1591	1577	[25]	2.7	0.30	-	-	2115	2121	[28]	2.7	0.24	-	-
**61**	caryophyllene oxide	1599	1595	[64]	6.0	0.10	0.5	0.03	1963	1960	[51]	5.8	0.54	0.3	0.12
**62**	muurola-4,10(14)-dien-1-β-ol	1622	1630	[25]	0.3	0.05	-	-	2259	-	*	0.1	0.15	-	-
**63**	dillapiole	1636	1620	[25]	0.8	0.01	-	-	2318	2327	[65]	1.2	0.22	-	-
**64**	*cis*-cadin-4-en-7-ol	1642	1635	[25]	0.2	0.01	-	-	2045	-	*	0.6	0.18	-	-
**65**	α-muurolol (=torreyol)	1658	1644	[25]	0.4	0.01	-	-	2170	2173	[51]	0.1	0.20	-	-
**66**	α-cadinol	1661	1652	[25]	0.6	0.10	-	-	2180	2188	[66]	1.2	0.31	-	-
**67**	furanoeremophilane	1676	-	§	31.3	0.41	-	-	2054	-	§	28.3	4.00	-	-
**68**	cyperotundone	1721	1718	[67]	0.9	0.10	-	-	2375	-	*	1.2	0.31	-	-
**69**	cyclocolorenone	1761	1759	[25]	1.3	0.01	0.5	0.06	2298	-	*	1.3	0.08	0.6	0.26
**70**	xanthorrhizol	1766	1751	[25]			-	-	2547	-	*	0.1	0.05	-	-
**71**	eremophilone	1726	1734	[25]			0.5	0.06	2299	-	*	-	-	0.4	0.10
**72**	bakkenolide A	1845	-	§	17.6	0.34	5.0	0.36	2430	-	§	16.3	0.55	5.5	0.56
	monoterpene hydrocarbons				4.3		-					3.9		-	
	oxygenated monoterpenes				1.6		7.0					1.8		6.0	
	sesquiterpene hydrocarbons				17.5		-					18.1		-	
	oxygenated sesquiterpenes				61.9		6.5					58.1		6.8	
	other compounds				2.6		27.2					2.4		30.6	
	total amount				87.9		40.7					84.3		43.4	

^1^ Calculated linear retention index; ^2^ Reference linear retention index; % = percent amount by weight; σ = standard deviation; § = identified by ^1^H NMR and ^13^C NMR spectroscopy; * = identified by mass spectrum only; trace ≤ 0.1%; - = not detected; mw = molecular weight.

**Table 2:** Enantiomeric separations with 2,3-diacetyl-6-tert-butyldimethylsilyl-β-cyclodextrin column.

Enantiomers	LRI	Enantiomeric Distribution (%)	*e.e.* (%)
(1*S*,5*S*)-(−)-α-pinene	925	100.0	100.0
(1*S*,5*S*)-(−)-β-pinene	977	100.0	100.0
(*R*)-(+)-sabinene	1007	15.4	69.2
(*S*)-(−)-sabinene	1012	84.6	
(*S*)-(+)-α-phellandrene	1025	100.0	100.0
(*S*)-(−)-limonene	1051	Inseparable	-
(*R*)-(+)-limonene			
(*S*)-(+)-β-phellandrene	1071	100.0	100.0
(*S*)-(−)-terpinen-4-ol	1379	100.0	100.0

*e.e.* = enantiomeric excess.

## Data Availability

Raw data are available from the authors (C.C.).

## Supplementary Materials

The following supporting information can be downloaded at: https://www.mdpi.com/article/10.3390/plants12061323/s1, Figure S1. Chemical structures of furanoeremophilane (**67**) and bakkenolide A (**72**); Figure S2. EIMS spectrum of furanoeremophilane (**67**); Figure S3. EIMS spectrum of bakkenolide A (**72**); Figure S4. ^1^H NMR (500 MHz) spectrum of furanoeremophilane (**67**) in CDCl_3_; Figure S5. ^13^C NMR (125 MHz) spectrum of furanoeremophilane (**67**) in CDCl_3_; Figure S6. ^1^H NMR (500 MHz) spectrum of bakkenolide A (**72**) in CDCl_3_; Figure S7. ^13^C NMR (125 MHz) spectrum of bakkenolide A (**72**) in CDCl_3_.
