# Supplementary material for: The Leaf Essential Oil of Gynoxys buxifolia (Kunth) Cass. (Asteraceae): A Good Source of Furanoeremophilane and Bakkenolide A
Source: Plants (Basel). 2023 Mar 15;12(6):1323. doi: 10.3390/plants12061323 (PMC10053332; doi:10.3390/plants12061323)
Supplement: Supplementary file 1 [file plants-12-01323-s001.zip › plants-2213416-SI.pdf]

Supplementary material

The Leaf Essential Oil of *Gynoxys buxifolia* (Kunth) Cass. (Asteraceae): a Good Source of Furanoeremophilane and Bakkenolide A

Carolina Cumbicus, Omar Malagón, Nixon Cumbicus, and Gianluca Gilardoni *

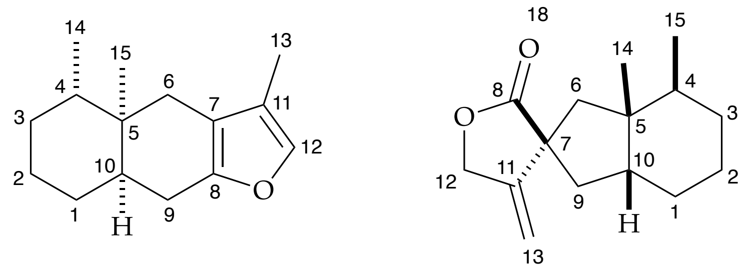

67 **72**

Figure S1. Chemical structures of furanoeremophilane (**67**) and bakkenolide A (**72**).

Furanoeremophilane (67). ^{1}H NMR [18] ($CDCl_3$, 60 MHz) δ(ppm): 0.90 (3H, s), 0.96 (3H, d, *J*=7.0 Hz), 1.91 (3H, d, *J*=1.1 Hz), 7.08 (1H, q, *J*=1.1 Hz). **^{13}C NMR [19]** ($CDCl_3$, 15 MHz) δ(ppm): 8.1 (CH_3), 15.2 (CH_3), 20.9 (CH_2), 23.9 (CH_3), 26.9 (CH_2), 28.9 (CH_2), 29.7 (CH_2), 29.7 (CH_2), 35.9 (C), 36.2 (CH), 36.6 (CH), 115.4 (C), 119.9 (C), 137.0 (CH), 148.8 (C).

Bakkenolide A (72). **^{1}H NMR [23]** ($CDCl_3$, 500 MHz) δ(ppm): 0.85 (d, 3H, *J*=6.8 Hz), 0.99 (s, 3H), 1.10-1.22 (m, 1H), 1.40-1.68 (m, 6H), 1.95 (d, 1H, *J*=14.2 Hz), 1.98 (dd, 1H, J_1=12.9 Hz and J_2=7.0 Hz), 1.98 (d, 1H, *J*=14.2 Hz), 2.09 (dd, 1H, J_1=13.3 Hz and J_2=12.9 Hz), 2.27 (dddd, 1H, J_1=13.3 Hz, J_2=7.0 Hz, J_3=4.9 Hz, and J_4=2.3 Hz), 4.74 (ddd, 1H, J_1=12.8 Hz, J_2=2.3 Hz, and J_3=2.1 Hz), 4.80 (ddd, 1H, J_1=12.8 Hz, J_2=2.3 Hz, and J_3=2.1 Hz), 5.03 (t, 1H, *J*=2.1 Hz), 5.11 (t, 1H, *J*=2.3Hz). **^{13}C NMR [23]** ($CDCl_3$, 100 MHz) δ (ppm): 16.4 (CH), 19.2 (CH), 21.0 (CH_2), 23.3 (CH_3), 30.9 (CH_2), 33.9 (CH), 42.4 (CH_2), 44.0 (C), 46.2 (CH), 48.5 (CH_2), 49.9 (C), 70.4 (CH_2), 105.8 (CH_2), 150.4 (C), 182.6 (C).

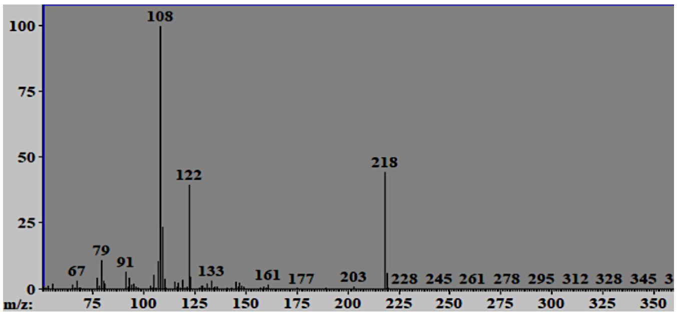

Figure S2. EIMS spectrum of furanoeremophilane (**67**).

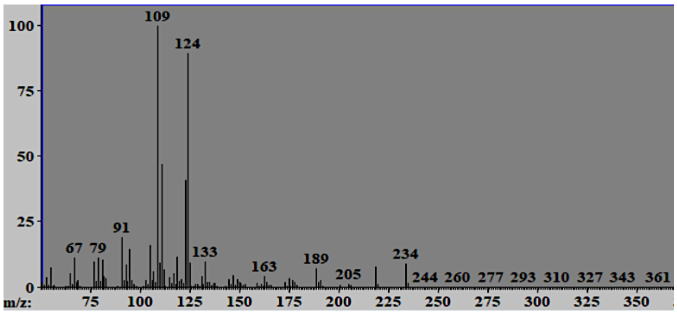

Figure S3. EIMS spectrum of bakkenolide A (**72**).

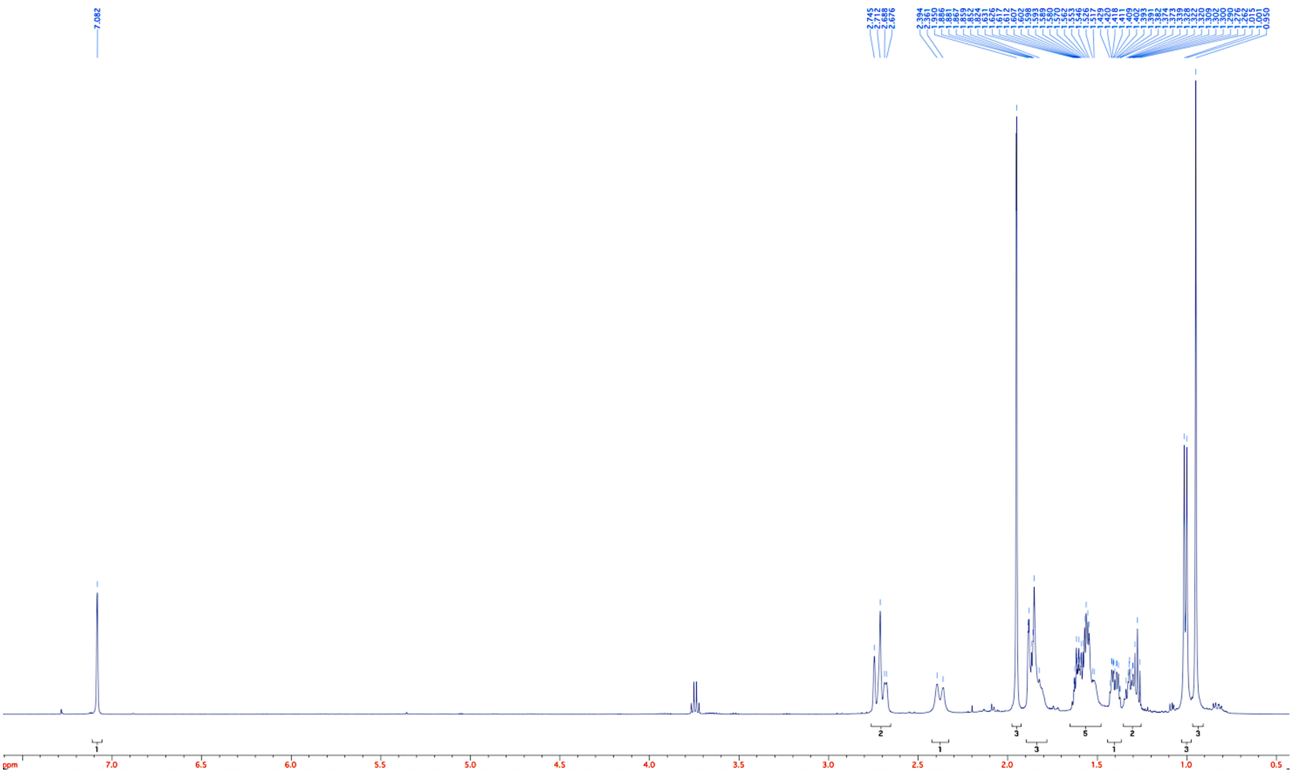

Figure S4. ^{1}H NMR (500 MHz) spectrum of furanoeremophilane (**67**) in $CDCl_3$.

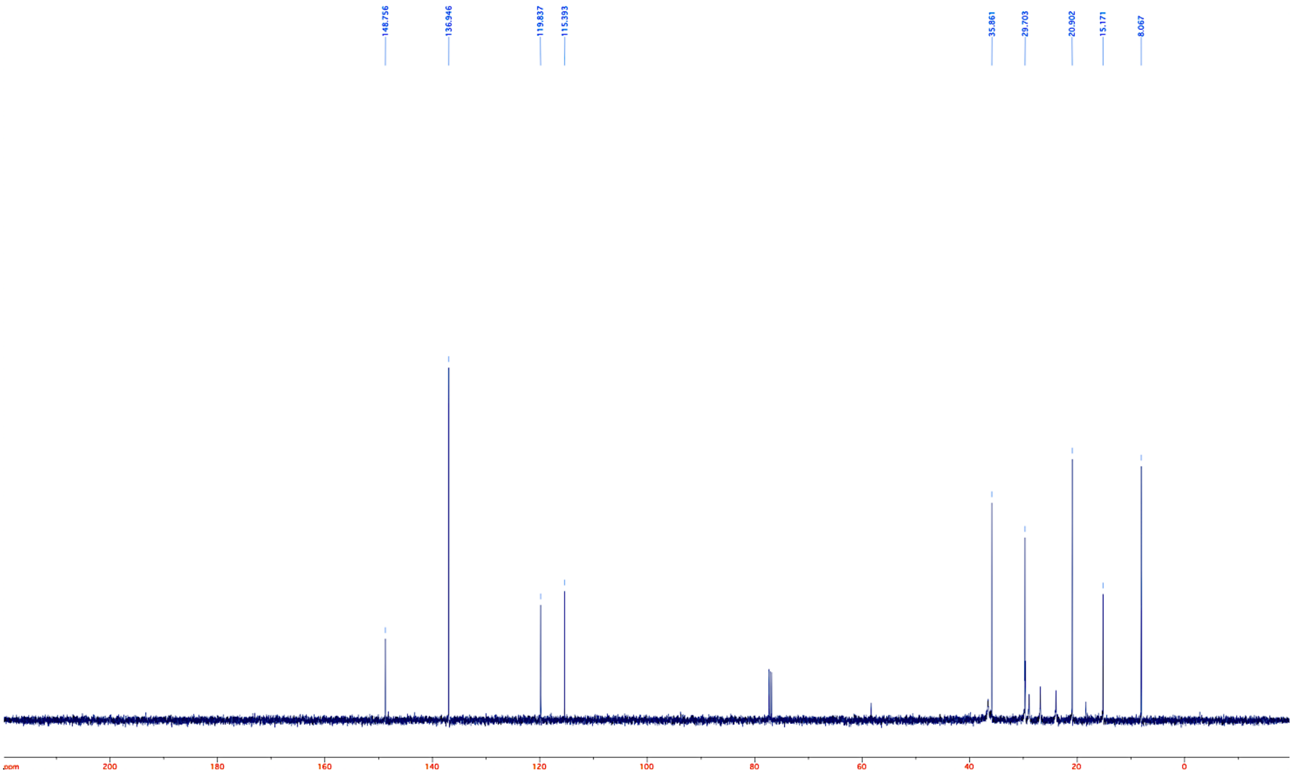

Figure S5. ^{13}C NMR (125 MHz) spectrum of furanoeremophilane (**67**) in $CDCl_3$.

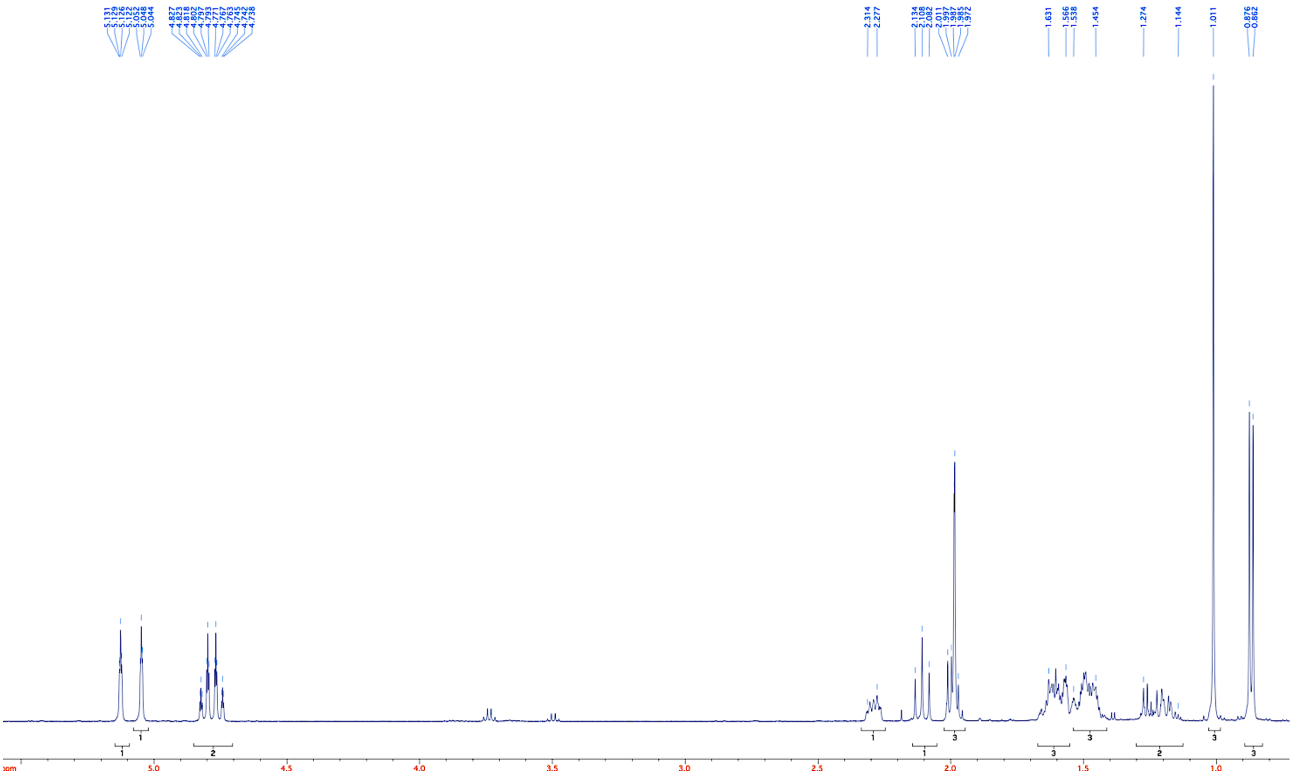

Figure S6. ^{1}H NMR (500 MHz) spectrum of bakkenolide A (**72**) in $CDCl_3$.

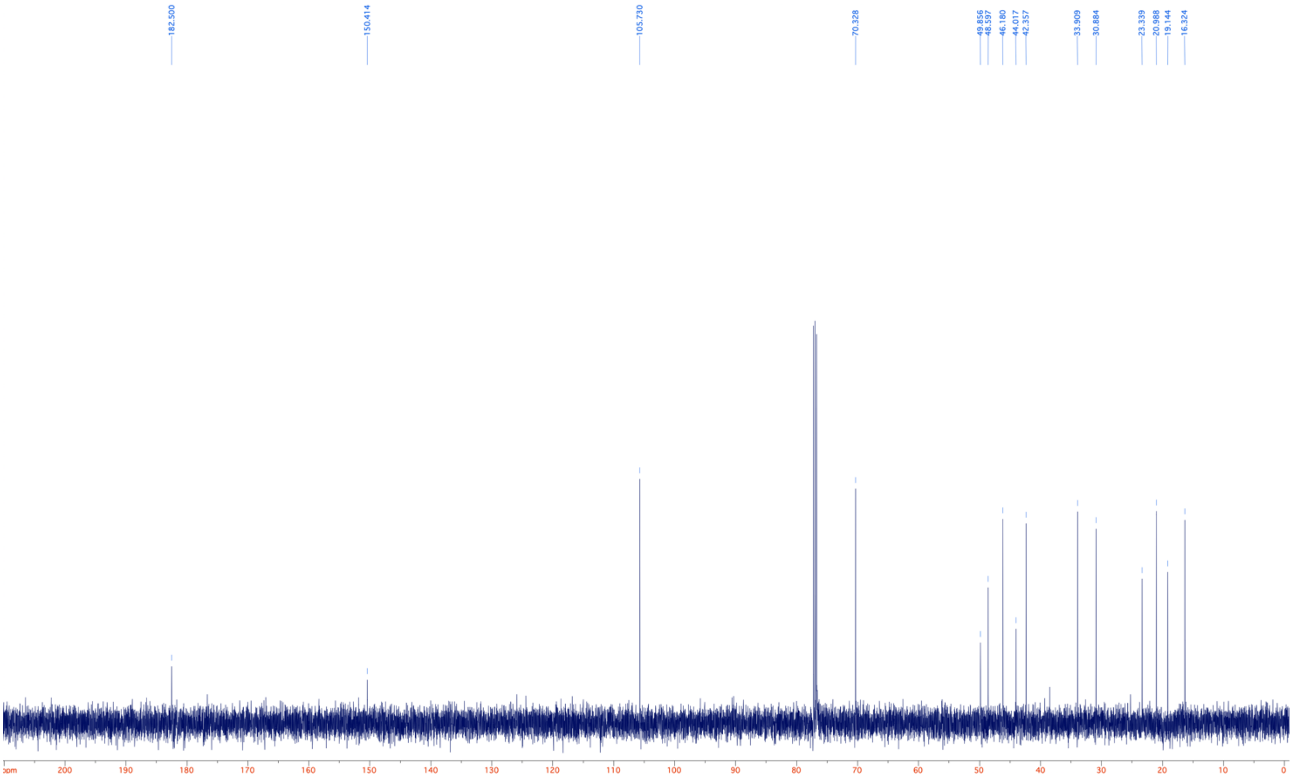

Figure S7. ^{13}C NMR (125 MHz) spectrum of bakkenolide A (**72**) in $CDCl_3$.
